# Nucleotide sequence of conjugative prophage Φ1207.3 (formerly Tn*1207.3*) carrying the *mef*(A)/*msr*(D) genes for eﬄux resistance to macrolides in *Streptococcus pyogenes*

**DOI:** 10.3389/fmicb.2014.00687

**Published:** 2014-12-09

**Authors:** Francesco Iannelli, Maria Santagati, Francesco Santoro, Marco R. Oggioni, Stefania Stefani, Gianni Pozzi

**Affiliations:** ^1^Laboratory of Molecular Microbiology and Biotechnology, Department of Medical Biotechnologies, University of SienaSiena, Italy; ^2^Section of Microbiology, Department of Biomedical and Biotechnological Sciences, University of CataniaCatania, Italy

**Keywords:** prophage, ICE, Φ1207.3, Tn*1207.3*, Tn*1207.1*, *S. pyogenes*, macrolide resistance

## Abstract

Genetic element Φ1207.3 (formerly Tn*1207.3*) is a prophage of *Streptococcus pyogenes* which carries the macrolide eﬄux resistance genes *mef*(A)/*msr*(D) and is capable of conjugal transfer among streptococci. Complete nucleotide sequence showed that Φ1207.3 is 52,491 bp in length and contained 58 open reading frames (ORFs). A manual homology-based annotation with functional prediction of the hypothetical gene product was possible only for 34 out of 58 ORFs. Φ1207.3 codes for two different C-methylation systems, several phage structural genes, a lysis cassette (composed by a holin and a peptidoglycan hydrolase), and three site-specific resolvases of the serine recombinase family.

## INTRODUCTION

In *Streptococcus pyogenes,* the *mef* (A)/*msr*(D) pair of genes encoding eﬄux resistance to 14- and 15-membered macrolides is carried by a mobile genetic element originally described as a conjugative transposon which was called Tn*1207.3* (Santagati et al., 2003; Pozzi et al., 2004). This element was found to be 52.5 kb in size, and to contain a complete copy of Tn*1207.1*, a 7,244-bp defective transposon previously found to carry *mef*(A)/*msr*(D) in *S. pneumoniae* (Santagati et al., 2000). Integration of the element into the *S. pyogenes* chromosome occurred at a specific GA dinucleotide target site located into the *comEC* coding sequence, with integration producing a duplication of the GA site. Upon conjugal transfer to *S. pneumoniae*, chromosomal integration occurred in *celB*, the pneumococcal homolog of *comEC*, at the same insertion site of Tn*1207.1* (Santagati et al., 2003; Pozzi et al., 2004). A copy of Tn*1207.1* was also found integrated in the 58,761-bp genetic element Φ10394.4, described as a prophage integrated at the same GA site within the *comEC* coding sequence of an erythromycin resistant clinical strain of *S. pyogenes* (Banks et al., 2003, 2004).

Here we report the manually annotated DNA sequence of Tn*1207.3* which indicates that the element is in fact a prophage, identical to the right end of Φ10394.4. For this reason the element was renamed Φ1207.3.

## MATERIALS AND METHODS

### *Streptococcus pyogenes* STRAINS AND GROWTH CONDITIONS

2812A is an erythromycin-resistant italian clinical isolate containing Φ1207.3 (Santagati et al., 2003). Bacteria were routinely grown in tryptic soy broth or tryptic soy agar (Difco) supplemented with 3% horse blood and, where appropriate, in presence of 1 μg/ml erythromycin.

### PCR AND SEQUENCING

Long PCR fragments were obtained with *Takara LA Taq* (Takara) following essentially the protocol suggested by the manufacturer. Briefly, the 25-μl reaction mixture was in 1X LA PCR Buffer II Taq buffer and contained: (i) 2.5 mM MgCl_2_, (ii) 200 μM dNTPs, (iii) 10 pmol of each primer, (iv) 0.25 units of *Takara LA Taq*, (v) 1 μl of liquid bacterial culture. Thermal cycling profile was as follows: 1 cycle at 92∘C for 2 min, then 30 cycles at 50∘C for 10 s, 68∘C for 15 min, 92∘C for 10 s, and 1 cycle at 50∘C for 1 min and 68∘C for 20 min. A primer walking approach (Santoro et al., 2010) was used to sequence the PCR products. The Expand High Fidelity PCR System (Roche) was used to produce PCR fragments of about 1,000 bp in size which were used as sequencing starting template to confirm sequence on the other strand. Four primer pairs were used to amplify the four fragments, ranging from 10,358 to 16,223 bp in size, containing Φ1207.3 and its chromosomal junction fragments.

### DNA SEQUENCE ANALYSIS

DNA sequence analysis was performed with the software Artemis version 11^1^. Manual gene annotation was carried out conducting BLAST homology searches of the databases available at the National Center for Biotechnology Information^2^. Protein domains were identified searching the protein family database Pfam available at the Wellcome Trust Sanger Institute^3^. The nucleotide sequence of Φ1207.3 is assigned GenBank accession no. AY657002.

## RESULTS

### Φ1207.3 NUCLEOTIDE SEQUENCE

**FIGURE 1:**
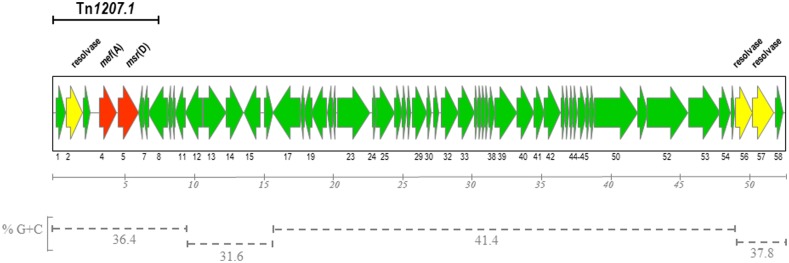
**Schematic diagram of *Streptococcus pyogenes* Φ1207.3.** The element is 52,491 bp in size and contains 58 open reading frames (ORFs). ORFs and their direction of transcription are represented by arrows and the annotated ORFs are indicated only by their numbers. Macrolide resistance genes and resolvase genes are reported as red and yellow arrows respectively. The region homologous to Tn*1207.1* is indicated by a solid bar. The different GC content of the various regions are indicated with dotted bars. Scale is in kilobases.

The complete nucleotide sequence of Φ1207.3 was obtained by primer walking on four long PCR fragments spanning the whole element. Inverse PCR on genomic DNA of *S. pyogenes* strain 2812A was carried out with divergent primers matching the already known 7,244-bp sequence of Φ1207.3 and its chromosomal junction sequences (Santagati et al., 2003). Amplicons obtained were used as sequencing template to determine internal sequences of Φ1207.3 on which primers were designed for the amplification of the four long overlapping fragments. The nucleotide sequence of Φ1207.3 was confirmed on the other strand using short PCR fragments as sequencing templates. Φ1207.3 was found to be 52,491 bp in length and DNA sequence analysis showed the presence of 58 open reading frames (ORFs), 44 of which have the same direction of transcription (**Figure 1**). Regions with different GC content can be could be identified in the DNA sequence of Φ1207.3: (i) the left end, containing ORFs 1–11, with 36.4% GC; (ii) ORFs 12–16 with 31.6% GC; (iii) ORFs 17–54 with 41,4% GC; (iv) the right end, corresponding to ORFs 55–58, with 37,8% GC (**Figure 1**). Comparison of Φ1207.3 with sequences present in public databases showed that: (i) the first 7,244 bp of the Φ1207.3 sequence are identical to the 7,244-bp element Tn*1207.1* of *S. pneumoniae* (Santagati et al., 2000, 2003); (ii) the whole Φ1207.3 is identical to the 52,491-bp right end sequence of *S. pyogenes* Φ10394.4 (Banks et al., 2003; Pozzi et al., 2004); (iii) Seven sequence fragments, ranging in size from 0.2 to 9.5 kb, show 76–97% nucleotide identity to the *S. pyogenes* prophage Φm46.1 (Brenciani et al., 2010); (iv) homologous sequence fragments are present in the genetic elements from the genomes of *S. dysgalactiae* AC-2713 (GenBank no. HE858529), *S. suis* JS14 (GenBank no. CP002465), and *S. agalactiae* A909 (GenBank no. CP000114).

### ORFs OF Φ1207.3

For 34 out of 58 ORFs it was possible a manual homology-based annotation with functional prediction of the hypothetical gene product (**Table 1**). Public protein databases and the Pfam protein family database were used for Blast searches of predicted gene products, taking into account significant homologies with functionally characterized proteins or good matches with Pfam domains. A putative ribosome binding sequence preceded all ORFs except *orf36* and *orf37.* The alternative start codon TTG was present in *orf34*, *orf36*, *orf43*, and *orf56*, whereas *orf37*, *orf38*, and *orf51* started with GTG. In the left arm of the element, the gene product of *orf2* was predicted to be a site-specific resolvase of the serine recombinase family, *mef*(A) (*orf4*) and *msr*(D) (*orf5*) encoded respectively the transmembrane domains and the ATP-binding domains of the ABC transporter responsible for the M-type resistance to macrolides (Iannelli et al., 2014, Abstract C1-1188, 44th Interscience Conference Antimicrobial Agents Chemotherapy, 2004), *orf8* and *orf11* were homologous to the Tn*5253 umuC*/*umuD* operon conferring UV resistance by activation of the SOS repair system (Munoz-Najar and Vijayakumar, 1999). SpyIM, encoded by *orf12*, is a C-5 cytosine-specific DNA methylase belonging to the SpyI type II restriction-modification cassette, and responsible for inhibition of restriction by SmaI, whereas *orf13* and *orf14* encoded the two subunits of SpyI restriction endonuclease (Euler et al., 2007). Other genes coding for putative restriction-modification proteins include *orf24* (restriction enzyme), *orf30* (endonuclease), *orf33* (cytosine-specific DNA methylase), while *orf29* gene product presented an S-adenosylmethionine synthetase domain which may act as a methyl group donor for Orf33. In the central region, *orf17, orf23,* and *orf25* encoded a DNA polymerase, a DNA primase, and a DNA helicase, possibly involved in phage DNA replication, whereas *orf38* and *orf39* encoded for the small and large subunit of the phage terminase, which, together with the portal protein encoded by *orf40*, could be involved in phage DNA packaging. *orf42*, *orf44*, *orf45,* and *orf50* code for putative structural phage proteins. A putative lysis cassette, which is typically composed by a holin and an endolysin (Young et al., 2000) was encoded by *hol* (*orf53*) and *skl* (*orf54*). At the right end of the element, *orf56* and *orf57* gene products were homologous to resolvases of the serine recombinase family, possibly involved in excision, circularization, and site specific integration of Φ1207.3, whereas *orf58* encoded a putative ADP-ribosyltransferase toxin.

**Table 1:** Annotated open reading frames (ORFs) of Φ1207.3.

ORF (aa)^a^	Annotation and comments (reference)	Pfam domains^b^ [*E* value]	Homologous protein ID/origin	Amino acid identity-similarity	*E* value^c^
*orf1* (218)	CpXC protein, contains four conserved cysteines forming two CpXC motifs	CpXC (15–138) [1.3e-31]
*orf2* (370)	Resolvase (Yang and Steitz, 1995)	Resolvase, N terminal domain (44–193) [5.4e-25] Recombinase (217–325) [2.9e-20]
*mef*(A)/*orf4* (405)	Macrolide ABC transporter, transmembrane domain	Transmembrane secretion effector (7–404) [1.7e-24]
*msr(D)/orf5* (487)	Macrolide ABC transporter, ATP-binding domain	ABC transporter, ATP-binding domain (20–86; 167–284; 312–437) [1.5e-6] [1.1e-7] [2e-17]			
*orf7* (122)	YolD-like protein, functionally equivalent to UmuD	YolD-like protein (28–111) [3.2e-12]			
*umuC/orf8* (471)	SOS responce UmuC protein (Munoz-Najar and Vijayakumar, 1999; Iannelli et al., 2014)		EU351020 orf70/Tn*5253 Streptococcus pneumoniae*	344/471 (73%) – 398/471 (85%)	0.0
*umuD/orf11* (229)	UmuD MucA homolog (Munoz-Najar and Vijayakumar, 1999; Iannelli et al., 2014)		EU351020 orf69/Tn*5253 S. pneumoniae*	110/230 (48%) – 161/230 (70%)	2e-57
*spyIM/orf12* (408)	C-5 cytosine-specific DNA methylase (Euler et al., 2007)				
*orf13* (555)	Restriction endonuclease subunit	AAA domain (dynein-related subfamily) (227–387) [9.1e-9]			
*orf14* (415)	Restriction endonuclease subunit (O’Driscoll et al., 2006)	LlaJI restriction endonuclease (13–383) [5.7e-120]	AAS99180/pNP40 *Lactococcus lactis*	157/406 (38%) – 251/406 (61%)	5e-81
*orf15* (384)	DNA binding, zinc finger domain protein	CGNR zinc finger (338–380) [4.2e-5]			
*orf17* (651)	DNA polymerase (Rådén and Rutberg, 1984)	DNA polymerase family A (247–520) [3.7e-14]			
*orf19* (190)	Conserved phage-associated protein	DUF2815 (11–188) [9.7e-65]			
*orf23* (761)	DNA primase (Yeo et al., 2002)	D5 N terminal like (285–472) [2.4e-29]			
*orf24* (100)	Restriction-modification enzyme, putative (Kinch et al., 2005)	VRR-NUC domain (8–88) [3.3e-16]			
*orf25* (458)	Helicase, putative	SNF2 family N-terminal domain (5–293) [8.9e-19]			
*orf29* (353)	*S*-adenosylmethionine synthetase (Takusagawa et al., 1996)	*S*-adenosylmethionine synthetase: N-terminal domain Domain (9–85) [6.9e-28] Central domain (105–214) [7.6e-30] C-terminal domain Domain (216–353) [1.2e-57]			
*orf30* (121)	HNH endonuclease, putative	HNH endonuclease (62–110) [1.6e-11]			
*orf32* (410)	DNA methylase	ParB-like nuclease domain (5–93) [1e-10] DNA methylase (189–381) [5.2e-29]			
*orf33* (388)	C-5 cytosine-specific DNA methylase	C-5 cytosine-specific DNA methylase (4–382) [1.5e-59]			
*orf38* (158)	Phage terminase, small subunit, putative	Phage terminase, small subunit (50–150) [8.2e-31]			
*orf39* (530)	Phage terminase, large subunit, putative (Schouler et al., 1994)	Phage Terminase (48–515) [1.1e-103]			
*orf40* (413)	Phage portal protein, putative (Moore and Prevelige, 2002)	Phage portal protein (30–381) [4.8e-121]			
*orf41* (228)	Clp protease, putative (Wang et al., 1997)	Clp protease (8–179) [4.5e-40]			
*orf42* (395)	Phage capsid protein, putative	Phage capsid family (124–393) [6.4e-78]			
*orf44* (109)	Phage head-tail adaptor, putative	Phage head-tail joining protein (7–105) [8.8e-15]			
*orf45* (126)	Tail component protein, putative	Bacteriophage HK97-gp10, putative tail-component (11–92) [1.2e-6]			
*orf50* (1039)	Tail tape measure protein, putative (Pedersen et al., 2000)	Phage-related minor tail protein (311–554) [2.7e-14]	AAG32164.1/*Lactococcus* phage TP901-1	152/693 (22%) – 280/693 (40%)	1e-22
*orf52* (967)	Host specificity protein (Duplessis and Moineau, 2001)		AAK83249/*S. thermophilus* phage DT1.2	113/418 (27%) – 186/418 (44%)	5e-40
*hol*/*orf53* (760)	Holin family protein (Lévesque et al., 2005)	Siphovirus protein of unknown function (DUF859) (1–516) [1.8e-214] Holin family (637–754) [4e-40]	AAW27943/*S. thermophilus* phage 2972	205/514 (40%) – 279/514 (54%)	8e-89
*skl/orf54* (260)	*N*-acetylmuramoyl-L-alanine amidase (Llull et al., 2006)	CHAP domain (13–142) [5.6e-10]	CAJ13672/*S. mitis* phage SK137	62/211 (29%) – 96/211 (45%)	9e-8
*orf56* (412)	Resolvase	Resolvase, N terminal domain (20–167) [3.8e-30] Recombinase (187–291) [4.2e-21] Recombinase zinc beta ribbon domain (307–368) [1.6e-8]			
*orf57* (521)	Resolvase	Resolvase, N terminal domain (28–176) [4.6e-33] Recombinase (200–306) [5.5e-24] Recombinase zinc beta ribbon domain (316–377) [2.4e-10]			
*orf58* (191)	ADP-ribosyltransferase toxin, putative	ADP-ribosyltransferase exoenzyme (66–181) [3.7e-17]			

*^a^The number of amino acids is shown in parentheses. ^b^The numbers in parentheses represent the part of the protein homologous to the Pfam domain. ^c^Method: compositional matrix adjust.*

## DISCUSSION

The complete and annotated DNA sequence of the mobile genetic element previously called Tn1207.3 clearly shows that the element is a prophage which we renamed Φ1207.3. At the sequence level, Φ1207.3 (52,491 bp) shows homology to two *S. pyogenes* prophages: (i) Φ10394.4 (58,761 bp), integrated at the same chromosomal site of Φ1207.3 (Banks et al., 2003; Pozzi et al., 2004); (ii) Φm46.1 (55,172 bp), integrated in the *rum* gene encoding an RNA uracil methyltransferase (Brenciani et al., 2010). The whole Φ1207.3 is identical to the right end of Φ10394.4, whereas high homology (>70%) to Φm46.1 is limited to 57% of the Φ1207.3 genome. Prophages similar to Φ1207.3 were also described by Giovanetti et al. (2005).

The recombination machinery of Φ1207.3 consists of three resolvases of the serine recombinase family. Serine recombinases are less common than tyrosine recombinases in prophage genomes and are usually present as a single large recombinase gene (Smith and Thorpe, 2002). Most of the *S. pyogenes* prophages present in sequenced genomes have a tyrosine recombinase (integrase) as the recombination module (Beres and Musser, 2007). The two resolvase genes at the right end of Φ1207.3 are probably transcribed as a single unit, as their coding sequences overlap by one nucleotide. It is likely that their gene products cooperate in mediating excision and integration of the Φ1207.3 DNA. This arrangement in tandem of two resolvase genes is also found in SSC*mec* elements (types I to IV) of *Staphylococcus aureus* (Wang and Archer, 2010), and in streptococcal prophage Φm46.1 (Giovanetti et al., 2005; Brenciani et al., 2010).

Since Φ1207.3 can move among streptococcal species (*S. pyogenes*, *S. pneumoniae*, *S. gordonii*) by a mechanism which fits the operational definition of conjugation (Santagati et al., 2003), this conjugative prophage may represent a novel class of genetic elements with a molecular mechanism of transfer that still needs to be elucidated. It is entirely possible that assembly of a complete phage particle may not be essential for the observed interspecific DNA transfer, and we hypothesize that the lysis cassette of Φ1207.3, rather than producing the bacterial cell burst after intracellular phage expansion (Young et al., 2000), could contribute to the formation of cytoplasmic bridges between donor and recipient cell.

## Conflict of Interest Statement

The authors declare that the research was conducted in the absence of any commercial or financial relationships that could be construed as a potential conflict of interest.

## Footnotes

1 http://www.sanger.ac.uk/Software/Artemis/website

2 http://www.ncbi.nlm.nih.gov/sites/gquery

3 http://pfam.sanger.ac.uk
